# Alveolar Ridge Preservation Revisited: A Multimodal Evaluation of Bone Preservation and Regeneration—Preliminary Findings from a Randomized Controlled Clinical Trial

**DOI:** 10.3390/bioengineering13040447

**Published:** 2026-04-11

**Authors:** Anja Heselich, Ramin Najafi, Sami Alammawi, Joanna Śmieszek-Wilczewska, Shahram Ghanaati

**Affiliations:** 1 FORM-Lab, Department for Oral, Cranio-Maxillofacial and Facial Plastic Surgery, Medical Center of the Goethe University Frankfurt, Goethe University, 60590 Frankfurt am Main, Germany; 2 Denticus Clinic, Lelewela 1/1, 44-100 Gliwice, Poland

**Keywords:** bone regeneration, CBCT, ridge preservation, histomorphometry, cavitations, covered socket residuum (CSR)

## Abstract

Alveolar ridge preservation using biomaterials is a well-established approach to counteract post-extraction bone resorption and optimize conditions for implant placement. However, most studies rely only on a single evaluation method and thereby risk overlooking essential aspects of alveolar regeneration. This preliminary analysis aimed to assess alveolar ridge preservation outcome using a multimodal approach combining histomorphometric, radiological, and image-based visualization methods. Twenty out of a planned 60 patients per group from an ongoing randomized controlled clinical trial were included. Patients were allocated to alveolar ridge preservation with a bone substitute material (BSM), a collagen-based material, a combination of both, or natural healing as control. Outcomes included CBCT-based volumetric analysis, histomorphometry, and primary implant stability via ISQ. Mineralized bone volume was significantly better preserved in bone substitute material groups compared to other groups, with BSM combined with collagen yielding the highest values. Histomorphometrically determined hard tissue fractions and implant stability were comparable across groups. Notably, CBCT-based visualization revealed non-ossified hypodense regions, so-called cavitations or covered socket residuum (CSR) within the former extraction sockets across all groups, independent of the biomaterial applied. BSM-based alveolar ridge preservation, particularly combined with a collagen membrane, most effectively maintained mineralized bone volume after extraction. Beside volumetric benefits, this preliminary in-dept analysis of the first part of the trial highlights cavitations/CSRs as a potentially underrecognized feature of post-extraction healing. Integrating quantitative with qualitative visualization-based assessments provides a more complete understanding of alveolar bone regeneration.

## 1. Introduction

Tooth loss can have various causes, with dental caries and periodontitis identified as the two main causes. With progress in aging, periodontitis in particular becomes the primary reason for tooth extraction [1,2]. Providing a significant impact on patients’ quality of life, especially due to functional impairments and psychosocial consequences [3,4], the goal must be an appropriate esthetic and functional dental rehabilitation following tooth loss. Various restorative protocols have been established for this purpose, with dental implants playing a particularly important role [5]. However, biologically active and mechanically stable bone is a prerequisite for clinically favorable outcomes [6], but the morphological changes the alveolar crest undergoes after tooth extraction, including structural and dimensional bone loss, make this task challenging [7,8]. The regeneration process can be further influenced by numerous external factors, such as the extraction technique, management of the extraction socket, and especially the patient’s regenerative potential [9].

The current state of knowledge regarding alveolar healing indicates a loss of dimension in bone tissue as well as scar formation in the soft tissue [10]. The degree of volume loss depends on individual patient factors and is primarily influenced by the position of the extracted tooth, the extent of trauma during extraction, the presence of infection, and previous periodontal disease [11]. In general, the alveolar ridge experiences greater resorption in the buccolingual width dimension compared to the vertical height dimension. Within six months after extraction, a horizontal reduction of up to 63% and a vertical volume reduction of up to 22% have been observed [12,13]. During and after tooth extraction, the therapeutic goal should be the preservation of an optimal implant site, both from a functional and esthetic perspective. To prevent atrophy of the extraction socket as much as possible and to ensure ideal conditions for subsequent implantation, various treatment strategies aimed at preserving the local bone volume to facilitate successful implantation with high osseointegration have been scientifically validated. Socket or alveolar ridge preservation are currently the most commonly employed method to counteract the bone dimensional loss. In this approach, bone substitute materials are placed into the fresh socket immediately after tooth extraction in order to promote new bone formation and to prevent alveolar atrophy [14,15,16]. However, the degree of effectiveness of socket preservation remains a subject of debate in the scientific literature, and bone atrophy continues to represent a significant clinical challenge [9,17,18].

Rather than attempting to fully prevent atrophy, the current approach often accepts this phenomenon and instead focuses on biomaterial-based regeneration of the atrophied socket or on comparative studies of treated versus untreated extraction sockets. There remains a strong need to better understand the physiological healing process of the socket. This understanding could facilitate the development of improved protocols to support bone healing and to minimize atrophy. Current research is particularly centered on the investigation of postoperative volumetric changes of the alveolar ridge following tooth extractions [19,20]. In addition, recent observations have identified non-ossified hypodense regions within healing extraction sockets, so-called cavitations or covered socket residuum (CSR), which may represent an underrecognized yet clinically relevant feature of post-extraction alveolar healing that warrants further systematic investigation [20,21]. Only a limited number of comparative histological studies are available, and only a heterogenous selection of studies with radiological evaluations can be found. The latter predominantly focuses on quantitative evaluations of bone change. The aim of our study was to achieve a deeper understanding of the bone regeneration processes post extraction and/or alveolar ridge preservation by combining quantitative radiological evaluations with histomorphometric and radiological-image-based visualizations. For this purpose, we applied the here-described multi-modal approach to gain first insights in a preliminary analysis of a controlled, randomized clinical trial on the effectiveness of alveolar ridge preservation.

## 2. Materials and Methods

### 2.1. Clinical Trial Study Design

#### 2.1.1. Study Design

The preliminary data described here are part of a multi-centered prospective, parallel-arm randomized controlled PMCF-clinical trial conducted in the Republic of Korea and Europe. The trial is registered at the German Clinical Trials Registry at https://www.drks.de/DRKS00031607. The European part of the trial is conducted under the supervision of the primary trial center at the University Clinics of the Goethe University, and the Denticus Clinic, Lelewela 1/1, 44-100 Gliwice, Poland as further trial center. Patients included in this publication were treated within the trial from January 2022 to May 2025.

Surgical interventions and follow-ups, performed by JS and SG, were approved by the Institutional Review Board of the Ethical Committee of the Medical Department of Goethe University (IRB approvals #2022/1078) and the Silesian Medical Council (#16/2023). The study followed the Declaration of Helsinki and national regulations in Germany and Poland for human studies. All participants provided written informed consent after being informed of the study’s procedures and objectives.

#### 2.1.2. Inclusion and Exclusion Criteria

Patients (≥18, ≤65 years) requiring premolar or molar extractions (excluding 3rd molars) and planned for dental implant therapy were recruited. Complete medical and dental histories were obtained. Exclusion criteria included lack of written consent, full mouth plaque score > 25%, severe systemic disease, smoking, alcohol or substance abuse, untreated periodontal disease, untreated caries lesions, acute abscess, pregnancy or nursing, or inability to follow study instructions.

#### 2.1.3. Sample Size

The sample size of the European part of the clinical trial was set to n = 240 patients in total, randomized to four groups of n = 60 per group, based on power calculation at a power of 80% and an expected drop-out rate of 20%. The data reported in this publication provide a pre-liminary insight into the ongoing trial, therefore the total number of cases is not reported. Patients included in this trial are patients selected in consecutive instances based on consecutive completion of bone volume analysis. Bone volume analysis was done in consecutive order of received data from these cases for evaluation. Same set of cases per group were selected for histological evaluation, visualization, and evaluation of implant-related parameters. For more details on case numbers per evaluation see Section 2.5.

#### 2.1.4. Randomization

Patients were randomly assigned to either of the four groups: (i) Control group (natural healing), (ii) Collagen group (socket filling with collagen plug or block), (iii) BMS group (alveolar ridge preservation with OCS-B^®^; NIBEC Ltd., Seoul, Republic of Korea), or (iv) BSM and with collagen membrane group (alveolar ridge preservation with OCS-B^®^ and cover of the socket with collagen membrane Regenomer^®^; both NIBEC Ltd., Seoul, Republic of Korea).

Based on the sample size calculation, computer-generated randomization (GraphPad Softwareversion 11.0.0, LLC) was performed by a study member uninvolved in treatment. Each patient was allocated to a sealed randomization letter, opened by the surgeon immediately before extraction.

#### 2.1.5. Follow-Up and Outcome Measures

Tooth extraction and treatment were assessed for bone condition and regeneration after tooth extraction and after a regeneration time of three months. The primary outcome was mineralized bone volume three months after extraction. The secondary outcomes were ratio of hard and soft tissue, and implant stability via ISQ.

### 2.2. Surgical Procedures

#### 2.2.1. Tooth Extraction and Alveolar Ridge Preservation

Extractions were performed under local anesthesia using a minimally traumatic technique without vertical releasing incisions. Sockets were inspected, curetted, and rinsed with sterile 0.9% saline. In the alveolar ridge preservation groups, sockets were either filled with collagen plugs or block (Regenomer^®^, NIBEC Ltd., Seoul, Republic of Korea), with BSM (OCS-B^®^; NIBEC Ltd., Seoul, Republic of Korea), or with the BSM and covered with a collagen membrane (OCS-B^®^/Regenomer^®^, NIBEC Ltd., Seoul, Republic of Korea). In all groups sockets were then sutured tension-free with non-resorbable horizontal mattress sutures. Control sockets remained untreated but received identical suturing.

#### 2.2.2. Materials

Bone substitute material OCS-B^®^ (NIBEC Ltd., Seoul, Republic of Korea), is a porous xenograft consisting of deproteinized bovine-derived hydroxyapatite cancellous granules, CE-marked (2012) and FDA-cleared (2013) for alveolar ridge preservation.

Collagen material Regenomer^®^ (NIBEC Ltd., Seoul, Republic of Korea) is an absorbable porcine-derived type I collagen wound dressing with a non-chemically crosslinked, macro- and microporous sponge-like matrix, FDA-cleared (2014) and CE-marked (2021) for application in/on tooth extraction sockets.

Implant selection was based on each patient’s individual clinical indication. Implants were sourced from three manufacturers: MegaGen (AnyRidge/AnyOne; MegaGen Implant Co. Ltd., Daegu, Republic of Korea), C-Tech (C-Tech Implant S.r.l., San Pietro in Casale, Italy), and Nobel Biocare (NobelActive; Nobel Biocare Services AG, Zurich, Switzerland). Implant diameters ranged from 3.5 to 4.5 mm and lengths from 7.0 to 11.5 mm.

#### 2.2.3. Follow-Up

In addition to clinical evaluation and photographic documentation, CBCT imaging was performed post-extraction and prior to implant placement at three months. During implant placement surgery, an initial pilot drilling was carried out at the intended implant site using a trephine drill to obtain a bone biopsy approximately 3 mm in diameter for histological analysis. Biopsy sampling was only performed when clinically feasible, i.e., when the planned implant diameter exceeded the trephine drill diameter; in cases where the implant diameter would be equal to or smaller than the trephine drill diameter, biopsy sampling would be omitted to avoid compromising implant stability and survival. Pilot drilling and, if applicable, biopsy harvest, was followed by sequential drilling in accordance with the implant system-specific protocols and the respective manufacturer’s instructions. Primary implant stability was evaluated via implant stability quotient (ISQ) measured using resonance frequency analysis directly after implant placement. An additional ISQ measurement was made two months after implantation.

### 2.3. Histology and Histomorphometry

#### 2.3.1. Biopsy Processing

Bone biopsies harvested during implantation surgery were fixed in 4% paraformaldehyde for at least 24 h, followed by decalcification in 10% EDTA solution (pH 7.4) for a minimum of two weeks. The samples were then incubated for defined periods in ascending ethanol concentrations, followed by incubation in xylene and finally embedded in paraffin. After paraffin embedding, histological sections of 3–4 µm were prepared, deparaffinized in xylene, rehydrated through descending ethanol concentrations, and subsequently subjected to standard hematoxylin and eosin staining.

#### 2.3.2. Histomorphometrical Analysis

Images of the total stained biopsy sections (“total scans”) were digitized using a Nikon Eclipse Ni-E microscope (Nikon, Japan) equipped with an automatic scanning table (Prior Scientific Instruments GmbH, Jena, Germany) and a DS-Ri2/Digital camera connected. Images were recorded in 100× magnification using NIS-Elements software (Nikon, Tokyo, Japan; v.BR 5.21.03). Total scans were then transferred to Fiji (Fiji Is Just ImageJ; v2.15.0 [22]). Scaling was transferred based on image scale bar. Total area, area of bone tissue, residual bone substitute material, and connective tissue were measured. Further, if applicable, regions of tissue artifacts were measured as well. Values for each sample were calculated as per cent values related to total scan area.

### 2.4. Radiological Evaluations

#### 2.4.1. Software and Data Format

The imaging software Fiji (Fiji Is Just ImageJ; v2.15.0) served as the foundation for the image-based evaluation of mineralized bone volume. Fiji is built on ImageJ2, an advanced version of the scientific and medical image analysis software ImageJ provided by the National Institutes of Health (NIH, Bethesta, MD, USA; imagej.nih.gov) [22]. All processing and analyzing tools used and implemented in the analysis macro are included in Fiji.

3D cone beam computed tomography (CBCT) images used for analysis had been recorded and analyzed in DICOM format.

OsiriX MD (Pixmeo SARL, software version UDI-PI:14.1.1, Bernex, Switzerland) with Meshmixer tool (Version 3.5.474), and Photoshop (Adobe Systems Software Version 26.3.0, Dublin, Ireland) were used for visualization analysis for CBCT images.

#### 2.4.2. Volume Evaluation

Bone volume was assessed based on CBCT scans. CBCTs were subjected to a qualitative analysis prior to evaluation, and CBCTs with optical artifacts, such as overexposed areas from adjacent prosthetic restorations or incomplete alveoli, were excluded to ensure a reliable assessment. Volume measurement was performed as previously described [23] using a novel image-based evaluation method. In short: CBCT scans post extraction and after regeneration were processed in Fiji (Fiji Is Just ImageJ; v2.15.0, based on ImageJ version 1.54p) to generate stacks of optical slices in buccal to lingual/palatinal orientation. Within each slice the area of the extraction socket (post-extraction) and area of mineralized tissue within socket (after regeneration) were measured. Volume was determined by summing the cross-sectional areas of each slice and multiplying the result by the slice thickness. Final mineralized bone volume after three months was calculated as percent based on original socket volume post extraction.

#### 2.4.3. Visualization Analysis

Qualitative image analysis of bone regeneration following tooth extraction was based on CBCT scans acquired immediately post-extraction and after a defined three-month healing period. Image processing was performed as previously described [20]. In brief, CBCT data were processed using OsiriX MD (Pixmeo SARL, software version U-DI-PI:14.1.1, Bernex, Switzerland) with a three-dimensional surface rendering tool to generate 3D representations of the jaw, including the region of interest (ROI) corresponding to the extraction site. Following 3D rendering, threshold adjustment and removal of noise artifacts, hemi-sections of the ROI were created. Further processing in Photoshop involved pseudo-coloring and overlaying of pre- and post-regeneration structures, allowing for visualization of newly formed bone, areas of bone loss, and cavitations corresponding to non-regenerated regions within the former extraction socket.

### 2.5. Statistics

#### 2.5.1. Number of Cases Evaluated

Evaluation of the mineralized alveolar volume and primary implant stability quotient (ISQ) was performed in n = 20 cases per group (n = 10 premolars/n = 10 molars); n = 80 in total. Histomorphometrical analysis was performed in n = 20 cases per group in Ctrl, BSM, and BSM with collagen membrane groups. In collagen group n = 19 (n = 9 premolars/n = 10 molars) were analyzed. One patient had to be excluded because biopsy harvest was not possible.

#### 2.5.2. Statistical Considerations and Tests

Descriptive statistics providing mean values per endpoint ± standard deviation were provided where indicated. Prior to inferential analysis, the assumptions of normality and homogeneity of variance were assessed using the D’Agostino and Pearson and/or Shapiro–Wilk tests, respectively. One-way ANOVA with Tukey’s post hoc for multiple comparisons at a confidence interval (CI) of 95% and an alpha of 5% was performed for statistical evaluation of mineralized alveolar volume analysis and primary implant stability (ISQ). Two-way ANOVA with Tukey’s post hoc for multiple comparisons at a confidence interval (CI) of 95% and an alpha of 5% was applied to histomorphometrical analysis, with treatment group and tissue type as the two independent factors.

## 3. Results

### 3.1. Mineralized Bone Volume

**Figure 1:**
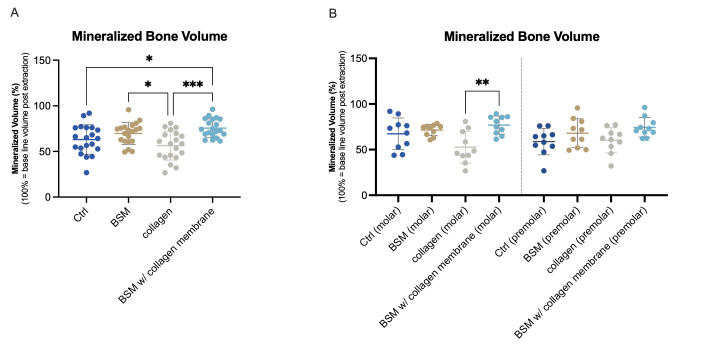
Mineralized bone volume three months post extraction. Mineralized bone volume was evaluated by image-based analysis of CBCTs scans post-extraction (defined as baseline (100% volume) of each socket) and after regeneration. (**A**) Groups without mineralized bone substitute material (control (dark blue), collagen (gray) showed statistically significant lower values (control * *p*< 0.05; collagen *** *p* < 0.001) than the BSM with collagen membrane group (light blue). The BSM group without additional membrane (brown) showed significantly higher mineralized bone volume than the collagen group (* *p* < 0.05). (**B**) Analysis according to tooth type (premolar/molar) showed comparable tendencies. Statistically significant higher volume was observed for BSM with collagen membrane group (light blue) compared to collagen group (gray) (** *p* < 0.01). Data represent mean percentage of mineralized bone volume based on post-extraction socket volume ± SD. Single dots represent values of individual patients. N = 20 patients per group (**A**), respectively n = 10 per group and tooth type (**B**).

Bone regeneration was analyzed via mean mineralized bone volume evaluation, calculated as percentage based on initial volume of extraction alveola. Regeneration of more than 50% mineralized volume was observed in all groups (Figure 1A). Both groups with BSM showed higher values (BSM: 69.72 ± 11.84%/BSM with collagen membrane: 75.61 ± 10.10%) than any of the BSM-free groups (control: 63.09 ± 16.18% / collagen group: 56.43 ± 15.83%). Statistically significant higher volume compared to control and collagen group was achieved in the BSM and the BSM with collagen membrane group compared to the collagen group, and further in the BSM with collagen membrane group compared to the control (Figure 1A). Furthermore a lower variation between the individual patients was found in the groups with BSM compared to the groups without. Separate analysis according to molar and premolar teeth showed comparable results with highest volume in the BSM with collagen membrane group (Figure 1B). However statistically significant differences were observed only between the BSM with collagen and the collagen group, with statistically significant lower values for the latter (*p* < 0.01).

### 3.2. Histomorphometrical Analysis

**Figure 2:**
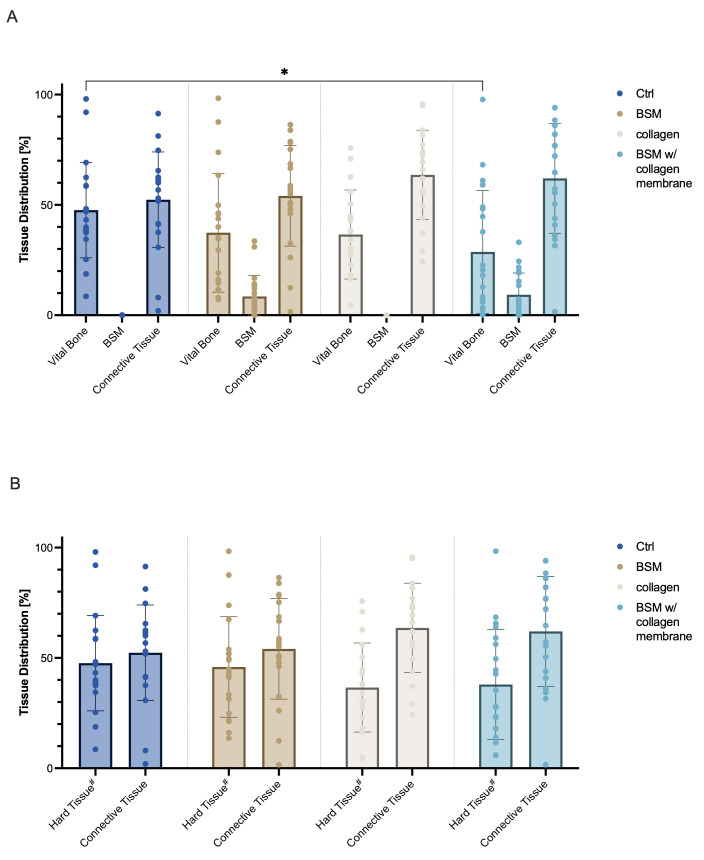
Histomorphometric analysis of tissue regeneration three months post extraction. Total scans of biopsies were histomorphometrically evaluated for distribution of vital bone, connective tissue, and (if applicable) residual bone substitute material (BSM). (**A**) Quantitative analysis of tissue distribution within biopsies taken from the planned implant bed showed comparable values for connective tissue, but less bone tissue in the BSM groups (brown/light blue) than the control (dark blue) group (control vs. BSM with collagen membrane: * *p* = 0.0132). Residual bone substitute material was comparable in the BSM with collagen membrane group (light blue) the BSM group (brown). (**B**) Mineralized structures (bone and BSM = “Hard Tissue”) were pooled for better comparison of all groups. No statistically significant differences were observed when comparing the pooled hard tissue. Data are represented as mean ± SD. Single dots represent values of individual samples. Baseline (100%) was defined as total scan area for each sample. N = 20 patients per group in all groups but collagen group (n = 19). #: Hard tissue = BSM + vital bone (BSM group/BSM with collagen group), or vital bone (ctrl group/collagen group).

Histomorphometrical analysis of biopsies taken 3 months after tooth extraction (Figure 2) showed comparable values regarding connective tissue with a mean area of 52.34 ± 21.65% of the total scan in the control group, 54.11 ± 22.79% in the with M group, 63.59 ± 20.24% in the collagen group, and 62.03 ± 24.90% in the BSM with collagen membrane group. However, vital bone fractions differed significantly (* *p* = 0.0132) between the control (47.66 ± 21.65%) and the BSM with collagen membrane group (28.72 ± 27.85%). No statistical difference was found regarding the vital bone values for the collagen group (36.57 ± 20.17%) and the BSM group (37.37 ± 26.94%) compared to any of the other groups. Mean area of residual bone substitute material, which could only be present in either of the BSM groups, was 8.52 ± 9.59% in the BSM group and 9.26 ± 9.84% in the BSM with collagen membrane group (Figure 2A). To allow for better comparability of mineralized structures between all groups, values for BSM and vital bone were pooled as hard tissue (Figure 2B). The mean area for hard tissue for BSM group was 45.89 ± 22.79% and for BSM with collagen membrane group 37.97 ± 24.90%. Comparison of the mean area of mineralized structures, respective hard tissue, did not show any statistically significant differences. In all groups a high variability between the samples can be observed. Individual analysis for both tooth types showed comparable values without any statistically significant differences.

### 3.3. Implants and Implant Stability

Distribution of implants placed were comparable according to length and diameter, with mean diameters between 3.97 and 4.105 and mean length between 9.25 and 9.675 (see Table 1).

All groups showed good primary implant stability quotients (ISQ) with mean values of approximately 70 and more (see Table 2). ISQ values showed tendencies to higher values in premolars compared to molars. No statically significant differences were observed between groups and/or tooth types.

### 3.4. Qualitative Evaluation and Cavitation Analysis

**Figure 3:**
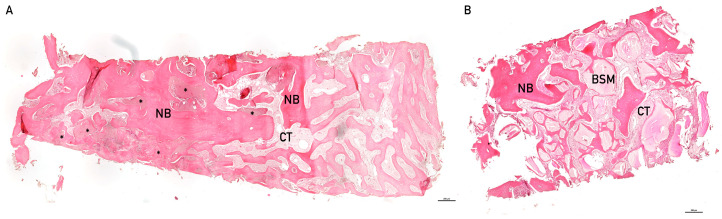
Representative examples of hematoxilin/eosine-stained biopsies. (**A**) Biopsy of socket preservation without BSM. Vital and new bone (NB) is visible throughout the sample. In between the vital bone structures, connective tissue (CT) can be observed. Asterisks (*) indicate examples of areas with tissue artifacts. (**B**) Biopsy with residual bone substitute material. Between vital bone and connective tissue residual bones’ substitute material can be observed (BSM), here visibly integrated in new bone structures, so called hybrid-bone.

**Figure 4:**
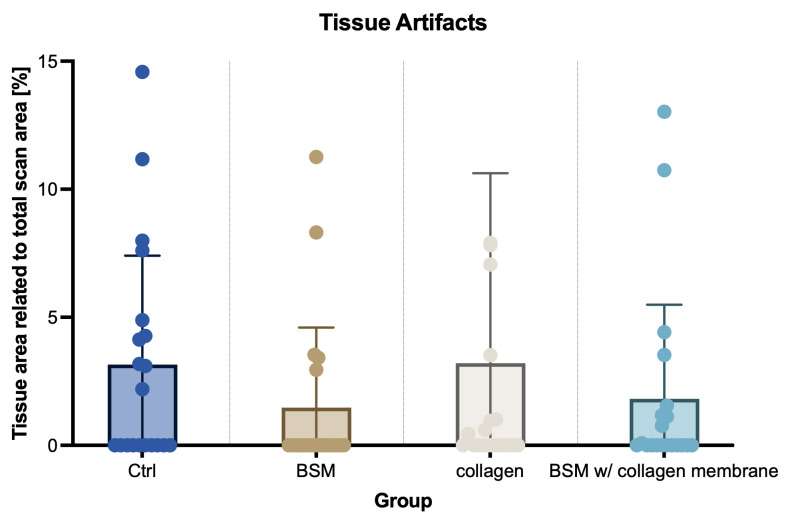
Quantification of observed tissue artifacts. Within each histomorphometric analysis evaluations were corrected for tissue artifacts not showing either of target tissue (vital bone/connective tissue/residual bone substitute material), but representing regions with degenerated tissue, corresponding to non-ossified hypodense regions found in so-called cavitations, also known as CSRs. Quantification of such regions showed lower occurrence and/or size in groups with BSM compared to groups without BSM. Data are represented as mean ± SD. Single dots represent values of individual samples. Baseline (100%) was defined as total scan area for each sample. N = 20 patients per group in all groups but the collagen group (n = 19).

The histomorphometric analysis revealed in some samples across all treatment groups, irrespective of augmentation, areas that were classified as degenerative tissue artifacts (Figure 3). The observed degenerative changes showed no association with the applied material, indicating that they occurred independently of material induction. There was even a tendency toward larger affected areas in groups without bone substitute material compared with groups receiving such material observed (Figure 4).

In the histological samples showing such tissue artifacts, differential staining did not allow for a definitive assignment of tissue type. In several areas, irregular and in part confluent regions with reduced or absent bone tissue were observed. These regions often appeared as loosely organized, cell-poor material. At some of these sites, loosening or degradation processes of the bone seemed to have occurred. However, inflammatory cell infiltration or a pronounced vascular component was not evidently present in these areas. The tissue architecture demonstrated degenerative alterations that are not characteristic of regular bone formation or active remodeling processes, which are typically composed of bone areas with differing structural organization. In multiple regions, fragments of lamellar, eosinophilic-stained bone with clearly recognizable structure were visible. The bony plates occasionally appeared fragmented and discontinuous at various sites. Some of such unstructured zones exhibited features that resembled structures occasionally observed in osteonecrotic alterations.

**Figure 5:**
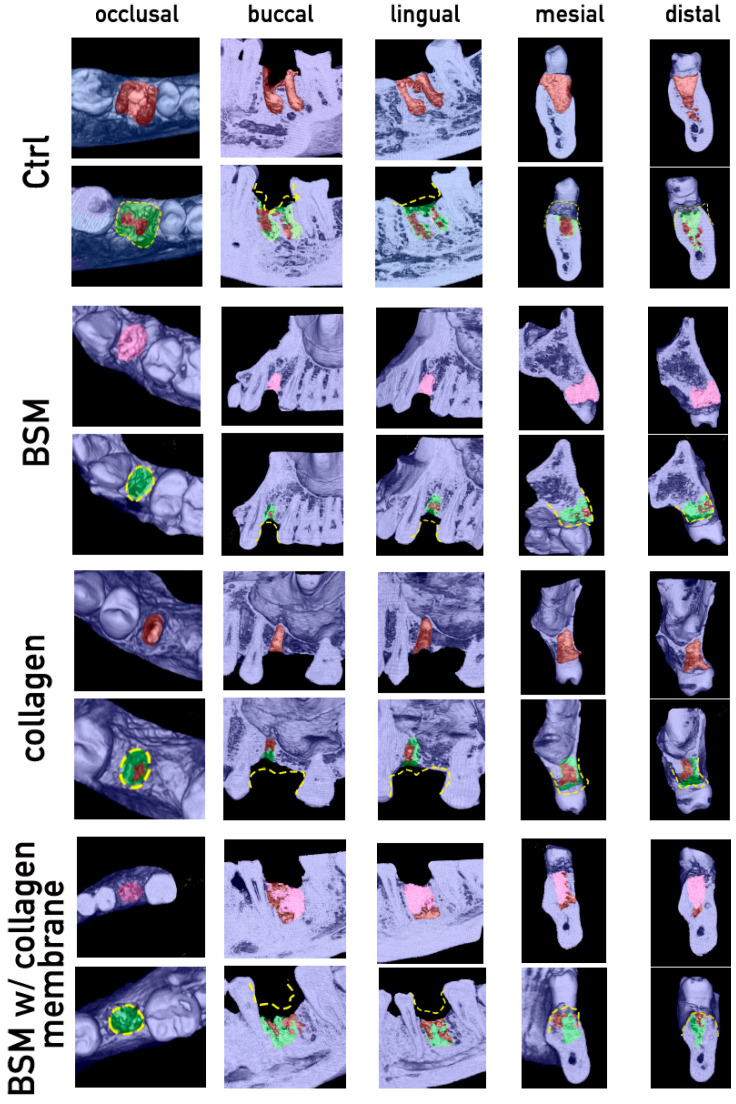
Cavitation analysis via visualization of CBCT imaging. Representative samples of cases with non-regenerated regions within the extraction alveola are shown. Visualization was done by selective image slicing of 3D CBCT images of the region of interest, respective former extraction sockets, and pseudo coloring of the different structures, as well as color-coded marking of morphological changes thereof. Cases of each group were qualitatively analyzed for degree of bone regeneration and occurrence of so-called covered socket residuum’s (CSRs), respective cavitations. The initial row within each group shows post-extraction/post-socket preservation situation in occlusal, buccal, lingual, mesial, and distal view. Second rows show the observed situation after 3 months regeneration time. In all groups bone regeneration to a certain degree can be observed (green); nevertheless, bone dimension loss (yellow dashed line) and non-regenerated regions (brown) became visible. Pseudo colors represent the following structures: blue = original bone/teeth; brown = unfilled/bone free regions; pink = regions filled with bone substitute material; green = regenerated bone structures.

Samples showing tissue artifacts were further qualitatively analyzed by generating three-dimensional visualizations of the corresponding CBCT imaging (Figure 5). Comparison of the post-extraction situation to the post-regeneration situation confirmed bone regeneration in all cases (Figure 5, green). Bone dimension loss was visible to a certain extent in all cases (Figure 5, yellow dashed lines), and further low-density regions representing insufficient regenerated regions (Figure 5, brown spaces within green regenerated zones).

## 4. Discussion

Although alveolar ridge or socket preservation techniques have been the focus of a broad variety of clinical trials for several years, evaluating different material compositions and combinations, as well as different clinical approaches, no gold standard for bone preservation following tooth extraction has yet been established. Further, with continuously emerging new materials the research in this area will be ongoing. The main goal will remain the achievement of sufficient bone and soft tissue supply for implant placement and counteracting any atrophic processes during the regeneration.

A major challenge in comparing results across trials is the variability in evaluation approaches, in addition to differences in clinical protocols such as varying time points, variable. While each provides interesting and valuable insights, comparing results from different methods remains challenging. Radiological methods like CBCT imaging provide non-invasive three-dimensional views of the clinical situation, whereas histological evaluations of bone biopsies from the augmented region allow for differentiation between tissue types. To achieve a deeper understanding of the bone regeneration processes post-extraction and/or alveolar ridge preservation we combined quantitative radiological evaluations with histomorphometric and radiological-image-based visualizations to gain preliminary insights by analyzing initial data from a randomized, controlled clinical trial.

When considering values of regeneration and volume preservation reported in the literature and in previous study analyses, one frequently encounters values that were back-calculated on the basis of height loss and width measurements [7,24]. Reliable data that are currently used as reference values in the field of post extraction regeneration have therefore been extrapolated from linear changes and can consequently capture only superficial changes within the alveolus. Deeper alterations obscured in two-dimensional radiograph or overlooked when exclusively linear measurements are applied may therefore have contributed to the variability in reported results. To overcome this issue we recently developed a semi-quantitative image-based approach for analyzing the mineralized volume within a former extraction socket, which allows for non-invasive evaluation of preservation and/or regeneration efficacy [23]. Within the quantitative evaluation of the first 20 of a total of 60 patients per group of the clinical trial, we observed a statistically significantly better-preserved mineralized volume in groups with BSM-based alveolar ridge preservation compared to control or collagen-only preserved sockets. In particular, the common approach to combine BSM with a collagen membrane demonstrated the highest values of preserved mineralized bone volume. This is well in accordance with conclusions of a recent meta-analysis by Lopez-Valverde et al., indicating a clear trend toward greater preservation of alveolar height and width when bone substitute materials were used in combination with a resorbable collagen membrane [25].

The histomorphometric distribution of vital bone and residual material is regarded as an indirect predictor of osseointegration quality. In the present preliminary study, histological observations supported quantitative evaluations in terms of presence of sufficient mineralized tissue. The BSM group showed the highest proportion of residual bone substitute material, while the amount of vital bone was significantly lower compared to the control and collagen groups. These findings are in line with previous studies reporting lower bone formation but increased volume stability in xenogeneic materials such as DBBM [26,27,28]. Although current evidence from meta-analyses does not conclusively demonstrate that the mere presence of residual bone substitute material directly contributes to increased implant stability [29], histological and composite bone studies indicate that implants can osseointegrate within a complex mixture of new bone and residual graft particles [30]. The residual BSM observed here, especially if integrated and forming a hybrid structure of BSM and new bone structures, may provide mechanical stabilization for subsequent implantation. Therefore, the total amount of residual BSM and new bone should be considered as the total hard tissue volume. When comparing this with the viable bone tissue observed in BSM-free groups, comparable hard tissue structures were found across all groups.

Focusing on individual cases of the early-stage assessment, a high interindividual variability was observed, particularly in the BSM and control groups. This variability may be explained by biological and clinical factors such as age, systemic diseases, extraction morphology, or surgical technique. Methodological aspects of biopsy sampling may also have influenced the results.

Based on the assumption that bone volume and quality are critical to prevent implant failure [31] one would expect significant differences in primary implant stability when differences in bone volume are observed. Trisi et al. demonstrated in an animal model that a higher proportion of vital bone correlated with improved biomechanical implant stability [32]. Nevertheless, despite significant differences in bone formation, resonance frequency analysis (ISQ) did not reveal group differences in the initial evaluation of the present study. A similar pattern was observed for the amount of hard tissue observed in bone biopsies, where no statistically significant differences were detected in the analyzed cohort. However, the biopsies in the analyzed clinical trial were dependent on implant position planning. From an ethical perspective, it would not have been justifiable to compromise the newly regenerated region by obtaining a biopsy from the augmented site when, based on the patient’s individual clinical situation, implant placement had to be planned in a more lateral position to achieve adequate implant stability. Consequently, biopsies were harvested at the planned implant position.

It must be emphasized that a single biopsy retrieved from the planned implant site represents only a spatially limited sample of the entire regenerated socket. Bone regeneration within an extraction socket is known to proceed in a spatially heterogeneous manner, with potentially substantial regional differences in tissue composition between central, peripheral, buccal, and lingual areas. As a result, a single-point biopsy carries an inherent risk of not being representative of the overall regenerative outcome. This spatial sampling bias has a direct impact on how histomorphometric results should be interpreted, since the observed tissue composition may reflect local conditions at the implant site rather than the regenerative status of the whole socket. This limitation is particularly relevant when comparing any histomorphometric findings across groups, as biopsy positions may systematically differ due to individual implant planning decisions.

This carries the considerable disadvantage that in samples with a less optimal bony situation the implant stabilization would rely on residual bone. This provides an unwanted bias, e.g., leading to the observation of more bone in samples without any preservation or efficient regeneration at all. Nevertheless, histomorphometric analysis is a recognized method for the quantitative evaluation of bone regeneration following augmentation procedures. The reliability of histomorphometric data, however, depends strongly on biopsy sampling and processing. Biopsies taken too buccally, apically, or in direct contact with residual biomaterial may lead to biased results because of regional differences in atrophy [7]. Inadequate cylinder diameter or insufficient depth of sampling also increase the risk of methodological error, especially in soft carrier materials, not only in dentistry but in bone biopsy procedures in general [33,34]. Thermal and mechanical artifacts, due to insufficient cooling, high drilling speed, or excessive axial pressure, may impair microscopic evaluation. Delayed fixation additionally promotes autolytic changes in cellular morphology. Such errors compromise both preclinical studies and clinical evaluation. Current guidelines therefore recommend standardized biopsy protocols, including defined sampling depth, internally cooled trephine drills, immediate fixation, and clear exclusion criteria for non-evaluable samples [35,36]. Furthermore, the suitability of common histological evaluations has been questioned, challenging the sole reliance on histological observations for assessing bone regeneration [37].

The observed primary implant stability results of our interims analysis are in contrast with expectations based on volumetric evaluation. Despite significant differences in preserved mineralized bone volume across groups, mean ISQ values were remarkably similar, ranging from 71.53 to 74.30 across all treatment groups, with overlapping standard deviations, and no statistically significant group differences. This suggests that beside well bone preservation and/or regeneration implant geometry and positioning are dominant factors for early osseointegration [38,39], independent of microscopically quantified tissue morphology. Notably, implant dimensions were comparable across all groups, with mean diameters ranging from 3.97 to 4.11 mm and mean lengths from 9.25 to 9.68 mm, suggesting that the absence of group differences in primary implant stability cannot be attributed to differences in implant geometry alone. Taken together, these findings underline that the relationship between bone regeneration quality and primary implant stability is more complex than volumetric parameters alone can capture, and may additionally be influenced by factors such as implant type, cortical anchorage, and surgical technique. Therefore, verification of alveolar ridge preservation efficacy in clinicals trial should not solely focus on implant-based parameters or histological findings in biopsies taken at implantation sites.

The combination of histomorphometry and three-dimensional imaging provides a complementary approach for evaluating bone regeneration and osseointegration. The integration of microscopic and macroscopic findings allows for a more complete understanding of tissue integration following augmentation procedures. Histomorphometry provides high-resolution, quantitative assessment of tissue architecture at the cellular level. It enables precise differentiation of vital bone, residual biomaterial, and intertrabecular connective tissue. Within the implant site, this allows conclusions to be drawn regarding bone quality, the remodeling behavior of biomimetic materials, and potential immunological responses, all of which are key for predicting osseointegration [32]. Radiological methods such as CBCT complement histomorphometric findings by providing three-dimensional information on bone density distribution, volumetric changes, and defect morphology, both for preoperative planning and postoperative follow-up [40].

The added value of combining both methods lies in the correlation of radiologically detected structures with histologically validated tissue findings. Radiology identifies macroscopic alterations such as bone collapse or hypodense zones, whereas histomorphometry differentiates between vital, regenerated, and pre-existing bone. Potentially insufficient or non-regenerated regions and residual material can thus be evaluated by both modalities and interpreted together.

Recent studies have increasingly adopted this combined approach. Peev et al., in a scoping review of 115 histomorphometric studies on dental implants, emphasized the importance of standardized protocols and the incorporation of radiological techniques for a comprehensive evaluation of implant integration [41]. Suh et al. demonstrated in an animal study that, combining micro-CT with histology, revealed significant advantages of 3D-printed implants compared to conventional designs in terms of early bone formation [42]. Jelušić et al. reported a histologically evaluated average bone formation of approximately 25% using a bovine bone substitute with hyaluronate in alveolar ridge preservation, while radiological follow-up confirmed stable hard tissue architecture [43].

A more recent observation in bone regeneration research focusing on alveolar socket healing is the occurrence of so-called cavitations or covered socket residuum’s (CSR) [20,21]. These phenomena describe non-ossified hypodense regions within the extraction socket. These were observed to varying extents in all four experimental groups in the early-stage analysis of the clinical trial presented here. Histologically, these presented as fibrovascular tissue or empty bone cavities, located both centrally and peripherally within the biopsies. Their random distribution does not suggest a causal relationship with the augmentation material. Patient-specific factors such as systemic influences, extent of extraction trauma, and postoperative mechanical loading may be more likely contributing factors. How such CSRs are formed and which factors predominantly favor their development remains to be explored in depth. Nevertheless, the latest observations on this topic from our group indicate that the post-extraction preparation of the extraction socket might have a major influence here [20,21]. In routine practice, comparable to the surgical protocol applied here, the post-extraction socket is cleaned by curettage and thoroughly rinsed; however this may not be sufficient to achieve a complete removal of infected or inflamed tissue within the socket. It is therefore reasonable to consider such residual inflamed tissue as the potential origin of the recently observed and described non-ossified hypodense regions, so-called cavitations or CSRs [21].

In a previous study we monitored bone healing in the maxilla for six months using cone beam computed tomography (CBCT) [44]. A significant loss of volume due to collapse of buccal and palatal bone walls was observed. Non-mineralized central areas frequently persisted, which were radiologically difficult to detect and were sometimes covered only by a thin osseous layer. These structures resemble the cavitations (hypodense regions) identified in the present preliminary study. Li et al. reported similar CBCT findings in maxillary anterior teeth, showing characteristic volume loss with central hypodense regions [45]. The inverted triangular morphology of the residual bone and the persistent absence of mineralized structures correspond to the cavitation phenomenon described here.

Comparison of the histological and radiological data presented here with previously described findings suggests that CSRs/cavitations may represent a physiological feature of alveolar remodeling, with their persistence and extent varying among individuals and potentially being influenced by the biomaterial applied. Complementary CBCT imaging in the present early-stage analysis of the clinical study confirmed the histological observations by demonstrating hypodense regions within the former socket in multiple cases. Based on these observations, it may be hypothesized that cavitations/CSRs could be clinically relevant, as they may compromise primary implant stability and serve as potential niches for bacterial colonization. Early detection through imaging might therefore be essential for treatment planning. The present findings indicate that cavitations should be considered as an independent parameter for evaluating augmentation outcomes, regardless of the biomaterial applied. Further, a novel approach termed guided open wound healing (GOWH) including a decortication step for more thorough preparation of the post-extraction socket and removal of potentially infected residual tissue prior to biomaterial application and/or wound closure, has been shown to achieve cavitation/CSR-free healing [21]. Improving the standard procedure in daily clinical practice by applying additional steps to optimize the socket treatment and thereby support subsequent regeneration should be taken into consideration in any socket or alveolar ridge preservation approaches [46].

## 5. Conclusions

Within the initial evaluation of the first third of cases of the underlying randomized controlled clinical trial, volumetric analyses confirmed superior preservation of mineralized bone volume in groups that underwent bone substitute material-based alveolar ridge preservation, with the combination of BSM and collagen demonstrating the highest preservation values. Despite these differences in bone volume and quality, no statistically significant differences in primary implant stability were detected, suggesting that implant type selection and positioning may exert a greater influence on initial stability than the regenerative protocol applied. These findings underline that volumetric preservation alone may not be a sufficient surrogate parameter for predicting implant success, and that a multiparametric evaluation approach is warranted.

Histological findings in the same cohort confirmed new bone formation in all groups, as well as the presence of residual bone substitute material, combined with new bone in the alveolar ridge preservation groups. The combination of quantitative CBCT-based volumetric analysis with histomorphometric evaluation and image-based three-dimensional visualization proved to be a valuable multimodal approach enabling the detection of findings that would have remained undetected by either method alone.

Most notably, non-regenerated hypodense structures, identified as cavitations or covered socket residuum (CSR), were observed across all experimental groups. Should these preliminary observations be confirmed in the full cohort, they may carry clinical relevance, as such structures could potentially compromise primary implant stability and may represent possible niches for bacterial colonization, which might in turn influence implant outcomes. Their observed presence across all groups, independent of the biomaterial applied, raises the hypothesis that cavitations/CSRs may represent an underrecognized feature of post-extraction alveolar healing with potential clinical significance. Pending further validation, their systematic assessment may warrant consideration as an additional outcome parameter in future augmentation studies.

From a clinical perspective, the present preliminary findings support the use of BSM-based alveolar ridge preservation for maintaining mineralized bone volume following tooth extraction. Furthermore, the results suggest that thorough debridement prior to biomaterial application may be a relevant factor in supporting complete socket regeneration and preventing cavitation/CSR formation, which is an aspect that warrants further investigation.

Although the present findings represent preliminary results from an ongoing trial, which constitutes a limitation of this study, they emphasize the importance of integrating advanced clinical radiological imaging with histological and histomorphometrical analyses for the assessment of bone preservation and regeneration. Furthermore, the results highlight the value of detailed qualitative evaluation, as performed through image-based visualization in the present study, for identifying insufficient regeneration and/or cavitation/CSR formation, which may be associated with subsequent complications and an increased risk of implant failure. As this manuscript represents a preliminary analysis of an ongoing clinical trial, no inferential conclusions are drawn from the current dataset. Final conclusions regarding the efficacy of the investigated ridge preservation procedures will be made upon completion of the full trial with the planned sample size of n = 240 patients. As data from the full cohort become available, these preliminary observations will require confirmation and further elaboration to establish evidence-based clinical recommendations.

## Figures and Tables

**Table 1:** Implant size distribution according to group. Length and size range of the dental implants showed comparable ranges between 3.5 and 4.5 mm in diameter and 7.0 to 11.5 mm in length. Data show implant size range from min to max for each group; n = 20 patients per group.

Implant Size	Control	BSM	Collagen	BSM with Collagen Membrane
**diameter**	3.5–4.3	3.5–4.5	3.5–4.5	3.5–4.5
**length**	8.5–10	7.0–11.5	7.0–10	8.0–11.5

**Table 2:** Primary implant stability (ISQ) evaluation, based on measurement of ISQ values in three positions (buccal, lingual, occlusal), was determined directly after implant placement. Data show mean ISQ ± SD; n = 20 patients per group (premolar and molar tooth pooled), or n = 10 patients per group and tooth type.

Tooth Type		Control	BSM	Collagen	BSM with Collagen Membrane
**pooled**	Mean ISQ	71.53	74.30	73.18	72.23
	SD	3.88	5.91	2.96	6.90
**premolars**	Mean ISQ	71.97	75.57	73.13	75.00
	SD	4.22	3.97	3.19	4.96
**molars**	Mean ISQ	71.10	73.03	73.23	69.47
	SD	3.69	7.37	2.89	7.68

## Data Availability

Data are contained within the article.

## Abbreviations

The following abbreviations are used in this manuscript:

BSM	Bone Substitute Material
ISQ	Implant Stability Quotient
CBCT	Cone Beam Computed Tomography
CSR	Covered Socket Residuum
GOWH	Guided Open Wound Healing
